# Small-Molecule Electron Acceptors for Efficient Non-fullerene Organic Solar Cells

**DOI:** 10.3389/fchem.2018.00414

**Published:** 2018-09-18

**Authors:** Zhenzhen Zhang, Jun Yuan, Qingya Wei, Yingping Zou

**Affiliations:** College of Chemistry and Chemical Engineering, Central South University, Changsha, China

**Keywords:** organic solar cells, efficiency, small molecule, fused ring, perylene diimide

## Abstract

The development of organic electron acceptor materials is one of the key factors for realizing high performance organic solar cells. Compared to traditional fullerene acceptor materials, non-fullerene electron acceptors have attracted much attention due to their better optoelectronic tunabilities and lower cost as well as higher stability. Non-fullerene organic solar cells have recently experienced a rapid increase with power conversion efficiency of single-junction devices over 14% and a bit higher than 15% for tandem solar cells. In this review, two types of promising small-molecule electron acceptors are discussed: perylene diimide based acceptors and acceptor(A)-donor(D)-acceptor(A) fused-ring electron acceptors, focusing on the effects of structural modification on absorption, energy levels, aggregation and performances. We strongly believe that further development of non-fullerene electron acceptors will hold bright future for organic solar cells.

## Introduction

Energy is the important foundation of human survival and economic development. With the rapid development of the global economy, the traditional non-renewable fossil energy such as coal, petroleum, and natural gas appears to be decreasing, and the burning of fossil fuels brings about greenhouse gases such as carbon dioxide and other chemical pollutants. At the background of energy crisis and environmental pollution, the development of clean and renewable energy has become the world's urgent requirements (Zhan et al., 2015). The emerging new energy sources include nuclear, solar, wind, hydro, and tidal energy. Among them, solar energy has the advantages of being clean, non-polluting, widely distributed, and non-exhaustive. It meets the requirements of sustainable development in the world today. There are three main ways to use solar energy: solar to thermal energy conversion, photoelectric conversion and photochemical conversion. Presently, the photoelectric conversion is one of the world focuses. The development of photovoltaic cells has become a promising solution for transforming solar energy into electricity. The first photovoltaic cell based on monocrystalline silicon materials was invented by Bell Laboratories (Chapin et al., 1954). Since then, the performance based on inorganic semiconductor solar cells began to get improved. However, the shortcomings of the complicated preparation process, high production cost, inflexibility in processing limited the preparation and application of large-area inorganic solar cells. On the contrary, organic solar cells (OSCs) have some merits of light weight, low cost, mechanical flexibility (Sariciftci et al., 1992; Li and Zou, 2008; Krebs, 2009; Li, 2011; Li et al., 2012; Heeger, 2014). More importantly, organic raw materials are abundant and the photoelectric properties can be modified by simple and feasible organic synthesis.

Nowadays, the typical OSCs active layers are bulk heterojunction (BHJ) structures, which are based on percolate and continuous electron donor (D) and electron acceptor (A) blend films. The working mechanism of OSCs is generally divided into four steps: (1) The active layer absorbs photons and then forms excitons (electron-hole pairs); (2) Exciton diffuses to D/A interface; (3) Exciton dissociates into free holes and electrons; (4) Free holes and electrons transport to the corresponding electrodes through the donor and acceptor channels, and subsequently are collected by electrodes. Finally, the photocurrent is formed in the external circuit (Lin and Zhan, 2014). To achieve high efficiency, an amount of small molecule/polymer donor materials have been developed, the power conversion efficiency (PCE) of fullerene OSCs had made a dramatic progress with values over 10% after decades of the tireless efforts by scientific community (Zhao et al., 2016b). However, the further development of fullerene-based OSCs encounters bottlenecks due to the inherent defects of fullerene derivatives, such as limited tunability of absorption and energy level, costly preparation and purification as well as poor stability.

In contrast to fullerene derivatives, non-fullerene acceptors (NFAs) can be modified by classical synthesis strategies, for example donor (D)-acceptor (A) conjugation, conformation locked and incorporation of functional groups, which is beneficial to adjusting crystallinity, electrical and optical properties. Although the first bilayered OSC is based on non-fullerene acceptor, the development of the electron acceptor lagged far behind of the donor materials in early studies (Kallmann and Pope, 1959). Early stage, rylene diimides derivatives, including perylene diimide (PDI) and naphthalene diimide (NDI), occupied the forefront of the non-fullerene materials. Before 2013, the PCEs were only about 1–3% (Bloking et al., 2011). After decades of mediocrity, Yao's group reported a novel acceptor (bis-PDI-T-EG), the performance achieved first breakthrough with PCE of 4.03% (Zhang et al., 2013). The second progress was the discovery of the ITIC, when blended with PTB7-Th, the device delivered a PCE of 6.8%, which is higher than 6.05% efficiency of PTB7-Th: PC_61_BM based devices (Lin et al., 2015b). This inspiring study showed that the performance of non-fullerene solar cells is expected to catch up or even be superior to fullerene based solar cells. In recent years, non-fullerene solar cells have once again revived and become a hot topic in photovoltaic researches (Liang et al., 2017; Zhang et al., 2018a). Currently, the highest efficiency has exceeded 14% for single-junction NF-OSCs and 15% for tandem NF-OSCs (Che et al., 2018; Zhang et al., 2018b).

To achieve high performance NF-OSCs, the primary factor to consider is the design and synthesis of acceptor materials. Generally, a promising acceptor should meet the following criteria:

- The acceptor should have complementary absorption with the donor as much as possible to increase photon utilization, which is beneficial for achieving high external quantum efficiency (EQE) and short-circuit current density (*J*_sc_). For example, to better match high performance narrow bandgap donors, wide or ultra-narrow bandgap acceptors should be designed. Narrow bandgap acceptors are likely to work well with wide or medium bandgap donors. In addition, the photocurrent can be formed by generation of excitons from both donor (channel 1) and acceptor (channel 2). Thus, apart from the complementary absorption with donors, the optical absorptivity of the NFA is also important (Nielsen et al., 2015; Cheng et al., 2018; Wadsworth et al., 2018).
- Besides the absorption, the energy levels matching with the donor material facilitates high open circuit voltage (*V*_oc_) and low energy loss (*E*_loss_). For fullerene OSCs, the highest occupied molecular orbital (HOMO) or the lowest unoccupied molecular orbital (LUMO) energy offset should be larger than 0.3 eV. However, recent investigations showed that the efficient exciton seperation and charge transfer for most non-fullerene system still can take place under less than 0.3 eV energy level offset (Liu et al., 2016b). Therefore, NF solar cells have unexpected potential in improving *V*_oc_ and decreasing *E*_loss_ through the appropriate regulation of the energy level of NFAs and donors.
- Another complicated but important factor is nanoscale interpenetrating morphology while blended with donor materials. The ideal morphology should be moderately aggregations. Too small aggregate domains will reduce the optical absorption and charge transport, but excessive aggregations will reduce the excitons separation efficiency and cause geminate recombination losses. Modulating the crystallinity of molecules by changing their conformations or structures seemed to be a useful strategy (Liu et al., 2014; Li et al., 2017a). Enhanced noncovalent forces, such as π-π stacking, van der waals, hydrogen-bonding interaction, help to increase the crystallinity and thus improve the carrier mobility. On the contrary, reducing inter- and intramolecular forces and twisting the conformation of molecules as well as introduction of side chains helps to improve solubility and reduce aggregations (Zhan and Yao, 2016).

Except for the above mentioned prerequisites, simple synthesis and low cost NFAs are beneficial for practical applications.

**Figure 1:**
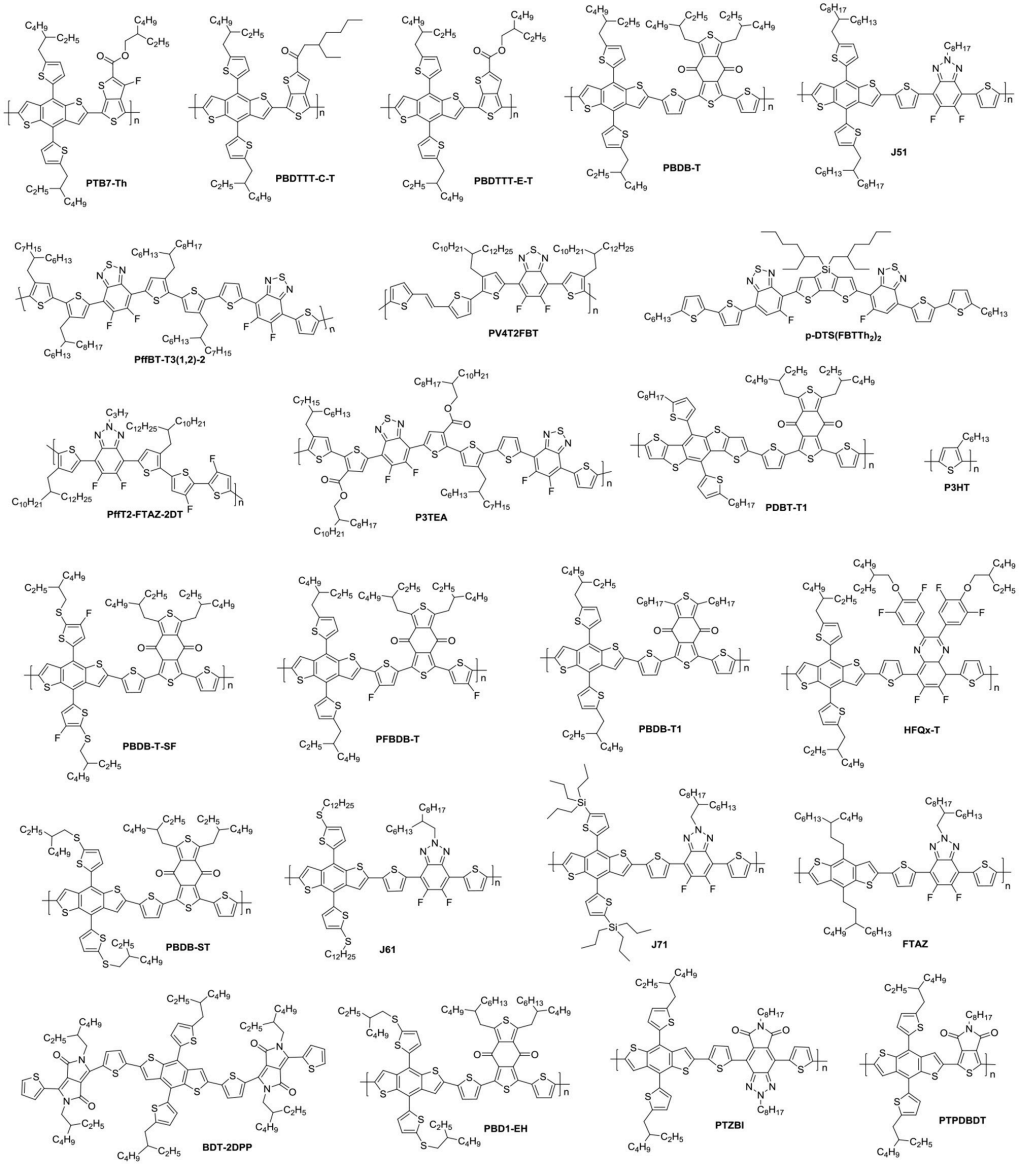
Chemical structures of the organic molecule/polymer donors referred in this review.

NFAs are classified into two major classes of polymers and small molecules. Small molecule NFAs have been intensively investigated by blending with polymer and small molecule donor materials (Figure 1) owing to their features over their polymeric counterparts, which include clear molecular structures, high purity and batch-to-batch stability (Roncali, 2009). In this review, we will focus on discussing the small molecule NFAs developed for high efficiency OSCs in recent years. Figure 1 listed chemical structures of the polymer donors referred herein.

## PDI based small molecule electron acceptors

PDI derivatives have attracted considerable attention as NFAs since they possess excellent optical absorption, similar energy levels to fullerenes, high electron mobility as well as good stability. Moreover, these properties can be easily tailored through the substituent groups on the bay region or on the nitrogen atoms (Zhao et al., 2013). The major problem is that PDI units tend to form large aggregates domains, which is more than the exciton diffusion length, led to less exciton separation and poor performance (Zhang et al., 2012). Therefore, it's essential to design and synthesize high performance PDI derivatives with moderate aggregations for effective exciton separation and charge transport.

**Figure 2:**
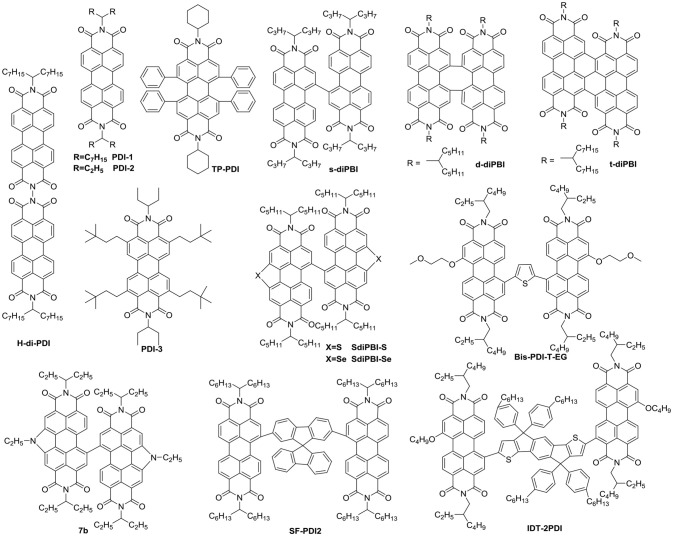
Chemical structures of selected perylene diimide-based electron acceptors from PDI-1 to IDT-2PDI.

Until now, several chemical modification methods have been used to reduce the self-aggregation of PDI and achieve good results (Figure 2 and Table 1). The initial design strategy is to introduce alkyl side chains on the nitrogen position or the ortho position. A series of alkyl-substituted PDI acceptor (PDI-1, PDI-2, PDI-3) was reported and studied, with improved solubility during solution processing and weakening the crystallinity to some extent. When blending with P3HT, the performance was poor (Kamm et al., 2011). But mapping other donors and optimizing the conditions for device fabrications, the OSCs based on p-DTS(FBTTh_2_)_2:_ PDI-2 blend film showed the PCE of 5.13% (Chen et al., 2015). TP-PDI was a bay-substituted tetraphenyl functionalized PDI derivative, which suppressed the strong aggregation tendency due to steric hindrance effects. While blended with PTB7-Th, a PCE of 4.1% was achieved (Cai et al., 2015).

**Table 1:** Summary of the photophysical and photovoltaic properties of selected perylene diimide-based electron acceptors from PDI-1 to IDT-2PDI.

**Acceptor**	**Donor**	***E*_g_ (eV)**	**LUMO/HOMO (eV)**	**μ_e_ (cm^2^·V^−1^·s^−1^)**	***V*_oc_ (V)**	***J*_sc_ (mA cm^−2^)**	**FF (%)**	**PCE (%)**
PDI-1	P3HT	–	–	–	0.25	0.21	22	0.01
PDI-2	P3HT	–	–	–	0.48	1.49	35	0.25
PDI-2	p-DTS(FBTTh_2_)_2_	1.91	−3.82/−5.87	4.5 × 10^−4^ (S,B)	0.80	10.07	64	5.13
PDI-3	P3HT	–	–	–	0.45	2.05	31	0.29
TP-PDI	PTB7-Th	1.85	−3.82/−5.69	–	0.87	10.1	46	4.1
H-di-PDI	PBDTTT-C-T	–	−4.1/−5.9	8 × 10^−3^ (O,B)	0.76	9.5	46	2.78
s-diPBI	PBDTTT-C-T	2.08	−3.87/−5.95	3.21 × 10^−5^(O,B)	0.76	10.58	47	3.63
d-diPBI	PBDTTT-C-T	2.22	−3.79/−6.01	–	0.74	5.76	36	1.51
t-diPBI	PBDTTT-C-T	1.69	−4.09/−5.78	1.84 × 10^−4^(O,B)	0.46	5.77	51	1.36
SdiPBI-S	PDBT-T1	2.20	−3.85/−6.05	3.20 × 10^−3^(S,B)	0.90	11.98	66	7.16
SdiPBI-Se	PDBT-T1	2.22	−3.87/−6.09	6.40 × 10^−3^(S,B)	0.96	12.49	70	8.42
7b	P3TEA	–	−3.8/–	10 × 10^−7^(S,B)	1.13	11.03	61	7.55
Bis-PDI-T-EG	PBDTTT-C-T	1.88	−3.84/−5.65	3.9 × 10^−4^(O,B)	0.85	8.86	54	4.03
SF-PDI_2_	P3HT	2.00	−3.71/−5.71	7.1 × 10^−5^(S,B)	0.61	5.92	65	2.35
SF-PDI_2_	PffBT4T-2DT	–	−3.83/−5.90	1.80 × 10^−4^(S,B)	0.98	11.10	58	6.30
SF-PDI_2_	P3TEA	–	–	–	1.11	13.27	64	9.5
IDT-2PDI	BDT-2DPP	1.54	−3.83/−5.53	2.3 × 10^−6^ (S,B)	0.95	7.75	42	3.12

*S stands for the mobility measured by the space charge limited current (SCLC) method and O for the organic field effect transistor (OFET)method; N stands for the neat film and B for the blended film*.

Moreover, PDI dimers can also reduce their crystallization tendency. Two PDI units were brought together using hydrazine as a linker, giving H-di-PDI. The perylene units are oriented perpendicular to each other, alleviated the aggregation. A PCE of 2.78% has been achieved when PBDTTT-C-T was used as donor material (Rajaram et al., 2012). Wang designed three PDI dimers (s-diPBI, d-diPBI, and t-diPBI), with singly-linked, chiral doubly-linked, and graphene like triply-linked between two PDI units, respectively. Blended with PBDTTT-C-T, s-diPBI delivered the best photovoltaic performance up to 3.63%, which is the result of a flexible structure with a twist angle of about 70° (Jiang et al., 2014). Subsequently, s-diPBI was modified by inserting thiophene and selenophene units in the bay positions, affording two new acceptors (SdiPBI-S and SdiPBI-Se). Both acceptors have a more twisted configuration and higher LUMO energy levels due to big and loose outmost electron clouds of sulfur and selenium. Moreover, the selenium is more polarized than sulfur, which is helpful to improving intramolecular interactions and carrier mobility. Thus, SdiPBI-Se exhibited a higher performance with PCE of 8.42 vs. 7.16% for SdiPBI-S when blended with same donor PDBT-T1 (Sun et al., 2015; Meng et al., 2016b). 7b was obtained by incorporating nitrogen heteroatom in the bay position of PDI to further study the potential of bay-linked PDI dimers. By modulating the N-R functional group, the self-assembly of acceptor would be changed. When the alkyl chain of the bay position is ethyl, the device demonstrated a best PCE of 7.55% with P3TEA as donor. More significantly, N-annulation of the PDI derivative can be synthesized in gram scale without the need for purification using column chromatography (Hendsbee et al., 2016).

**Scheme 1:**
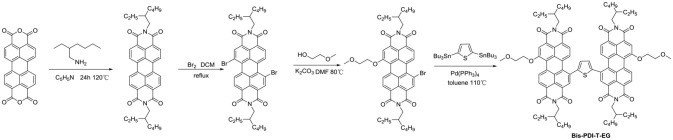
Synthetic route of Bis-PDI-T-EG.

Aside from direct linking two PDI units, twisted structure can be also achieved by using functional groups as the linkage. Bis-PDI-T-EG produced small phase domains with a size of ~30 nm. A promising PCE of 4.03% was obtained due to significant reduction of the aggregation (Zhang et al., 2013). This is the first time the PCE more than 4% in non-fullerene OSCs, demonstrated that the introduction of the π linkage is an effective method to improve photovoltaic performance, the synthetic steps of Bis-PDI-T-EG were shown in Scheme 1. Almost at the same time, another acceptor (SF-PDI_2_) featuring spirobifluorene linker was developed. When P3HT was used as donor, the PCE of 2.35% was achieved. The results demonstrated that steric-demanding substituents on PDI units was able to suppress self-aggregation and crystallization (Yan et al., 2013). Moreover, donor material PffBT4T-2DT can match particularly well with SF-PDI_2_ with complementary absorption and small driving force. The NF-OSCs possessed a high PCE of 6.3% (Zhao et al., 2015). After that, another NF-OSCs based on P3TEA: SF-PDI_2_ were fabricated, exhibiting ultrafast and efficient charge separation despite of a negligible driving force, with an excellent PCE of 9.5% (Liu et al., 2016b). A twisted PDI dimers (IDT-2PDI) with bulky indacenodithiophene as a bridge is developed as an electron acceptor. The OSCs based on BDT-2DPP: IDT-2PDI blend film showed a PCE of 3.12% (Lin et al., 2014a).

**Figure 3:**
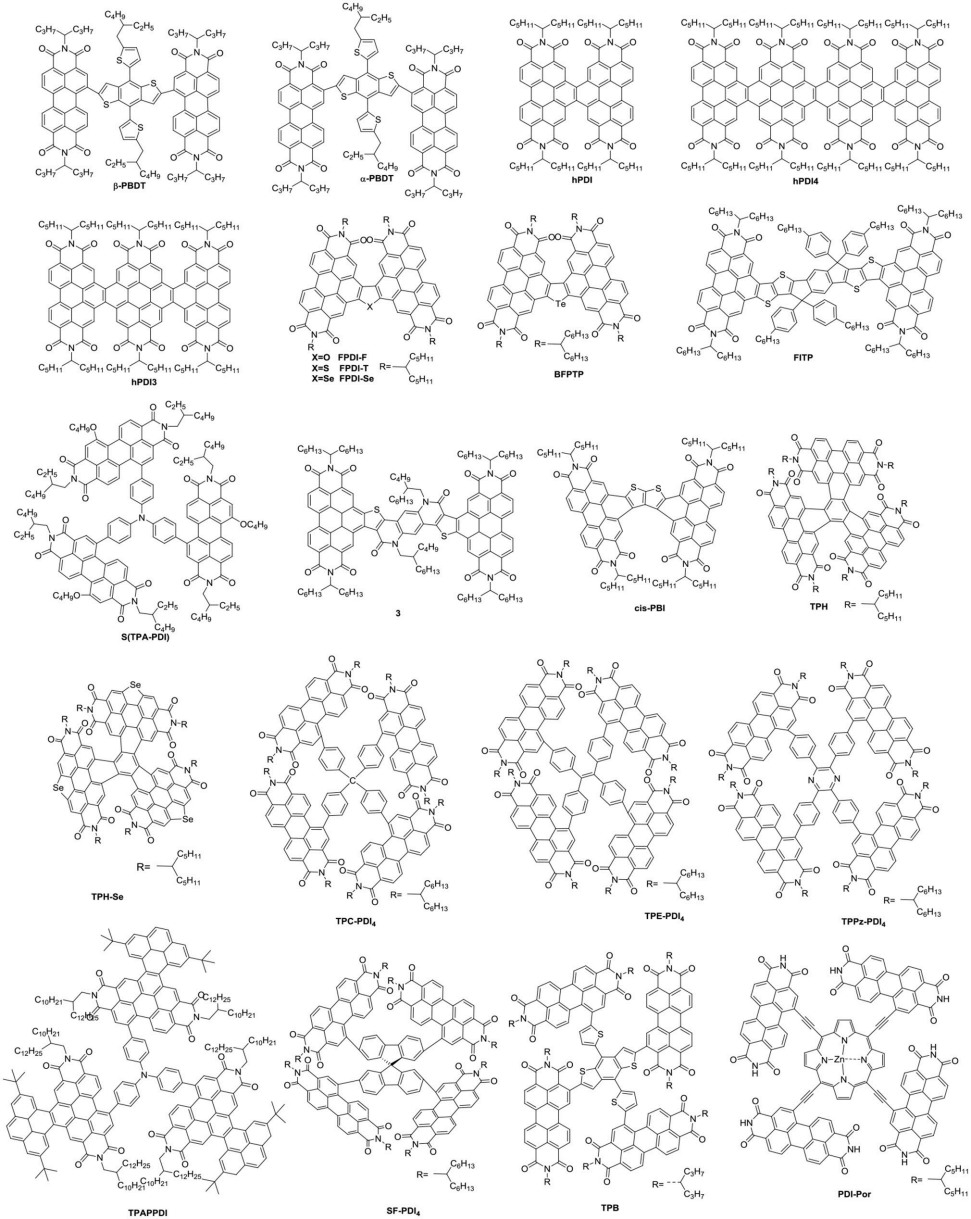
Chemical structures of selected perylene diimide-based electron acceptors from β-PBDT to PBI-Por.

Undoubtedly, both approaches to reduce the strong π-stacking aggregation by connecting two PDI units with single bond or linker have been efficient and shown improved photovoltaic performance, compared to traditional PDI derivatives. However, It must be admitted that the twisting of the structure will hinder the effective π-π stacking and diminish the charge transport. Thus, the trade-off between high electron mobility and effective exciton dissociation need to be solved in order to achieve excellent performance (Figure 3 and Table 2). Based on these considerations, two PDI dimers substituted at the α position (αPBDT) and β position (βPBDT) with benzodithiophene (BDT) unit were synthesized. The absorption revealed αPBDT have stronger intermolecular π-π stacking and higher packing order than βPBDT due to good planarity. The OSCs based on αPBDT as acceptor demonstrated a PCE of 4.92%, which is 39% higher than that of βPBDT counterparts, which is consequence of higher electron mobility and more efficient exciton dissociation in the αPBDT-based devices (Zhao et al., 2016a). A class of fused but helical PDI oligomers (hPDI, hPDI3, hPDI4) with ethylene group as bridges were designed and studied, which all possess strong light absorption, weak aggregation trendency and both hole and electron can be generated in both the donor and acceptor phases. The device based on PTB7-Th: hPDI4 reached a highest PCE of 8.3% (Zhong et al., 2014, 2015). A series of fused heterocycle PDI derivatives with different chalcogen atoms of O, S and Se (FPDI-F, FPDI-T, FPDI-Se,) were reported. Compared to unfused PDIs, fused PDIs increased effective conjugation and reduced reorganization energy helpful for high charge mobility, while maintaining nonplanar structure for suppress the strong aggregation. Moreover, the device based on FPDI-T showed a best photovoltaic performance with a PCE of 6.72% because of smallest twist angle leading to high packing order and close π-π stacking (Zhong et al., 2016). The first triplet tellurophene-PDI based acceptor (BFPTP) possessed long exciton lifetime and diffusion distances for efficient exciton dissociation rather than recombination. Thus, the PBDB-T: BFPTP blended films delivered a PCE of 7.52% (Yang et al., 2018a). A fused and twisted PDI derivative with twisted thieno[2,3-b]thiophene (TT) as linker (cis-PBI) was reported. When blended with PBDB-T, the OSCs demonstrated a high PCE of 7.6% as a result of high electron mobility and isotropic crystalline properties of electron acceptor (Jiang et al., 2017). A fused PDI derivative with indacenodithieno [3,2-*b*]thiophene (IDTT) as central core (FITP) maintaining rigid conjugated skeleton and hexylphenyl side chains of IDTT hindered the large crystallites. The devices based PTB7-Th: FITP exhibited a high PCE of 7.33% due to elevated LUMO and superior electron mobility (Li et al., 2016b). Compound 3 with a planar conformation utilized weak electron acceptor (thieno-pyride-thieno-isoquinoline-dione) bridge for the lateral PDIs. When blended with PTB7-Th, the devices delivered a PCE of 5.03% (Carlotti et al., 2018).

**Table 2:** Summary of the photophysical and photovoltaic properties of selected perylene diimide-based electron acceptors from β-PBDT to PBI-Por.

**Acceptor**	**Donor**	***E*_g_ (eV)**	**LUMO/HOMO (eV)**	**μ_e_ (cm^2^·V^−1^·s^−1^)**	***V*_oc_ (V)**	***J*_sc_ (mA cm^−2^)**	**FF (%)**	**PCE (%)**
α-PBDT	PTB7-Th	1.58	−3.78/−5.60	8 × 10^−4^(S,B)	0.81	12.74	46	4.76
β-PBDT	PTB7-Th	1.58	−3.76/−5.64	4.81 × 10^−4^(S,B)	0.81	9.80	44	3.49
hPDI	PBDTT-TT	–	−3.77/−6.04	3.4 × 10^−4^(S,B)	0.80	13.3	57	6.05
hPDI3	PTB7-Th	–	−3.86/−6.23	1.5 × 10^−4^(S,B)	0.81	14.5	67	7.9
hPD4	PTB7-Th	–	−3.92/−6.26	1.5 × 10^−5^(S,B)	0.80	15.2	68	8.3
FPDI-F	PTB7-Th	2.20	−3.80/−6.01	3.17 × 10^−7^(S,B)	0.94	8.79	40	3.29
FPDI-T	PTB7-Th	2.22	−3.77/−5.98	1.63 × 10^−4^(S,B)	0.94	12.28	59	6.72
FPDI-Se	PTB7-Th	2.22	−3.76/−5.96	1.21 × 10^−4^(S,B)	0.92	11.36	56	5.77
BFPTP	PBDB-T	2.12	−3.88/−6.00	3.06 × 10^−4^(S,B)	0.94	12.83	62	7.52
cis-PBI	PBDB-T	2.19	−3.74/−5.93	3.2 × 10^−3^(O,B)	1.00	11.9	64	7.6
FITP	PTB7-Th	–	−3.75/−5.48	3.66 × 10^−4^(S,B)	0.99	13.24	56	7.33
3	PTB7-Th	–	−3.05/−5.84	3.9 × 10^−6^(S,B)	0.94	12.12	46	5.03
S(TPA-PDI)	PBDTTT-C-T	1.76	−3.70/−5.40	3.0 × 10^−5^(S,B)	0.87	11.27	33	3.22
TPAPPDI	PBT1-EH	1.88	−3.59/−5.47	2.0 × 10^−3^(S,B)	1.21	6.83	62	5.10
TPH	PDBT-T1	2.19	−3.83/−6.02	1.5 × 10^−3^(S,B)	0.97	12.40	70	8.28
TPH-Se	PDBT-T1	2.17	−3.80/−5.97	2.2 × 10^−3^(S,B)	1.00	12.72	72	9.28
TPC-PDI_4_	PffBT-T3(1,2)-2	2.25	−3.75/−6.00	2.8 × 10^−4^(S,B)	1.04	8.8	61	4.7
TPE-PDI_4_	PffBT-T3(1,2)-2	2.05	−3.72/−5.77	1.2 × 10^−3^(S,B)	1.03	11.1	55	6.0
TPPz-PDI_4_	PffBT-T3(1,2)-2	2.10	−3.76/−5.86	2.5 × 10^−3^(S,B)	0.99	12.7	57	7.1
SF-PDI_4_	PV4T2FBT	2.05	−3.78/−5.97	1.93 × 10^−5^(S,B)	0.90	12.02	54	5.98
TPB	PTB7-Th	1.82	−3.89/−5.71	6.10 × 10^−6^(S,B)	0.79	18.20	59	8.47
PBI-Por	PBDB-T	1.48	−3.68/−5.46	1.0 × 10^−2^(O,B)	0.78	14.5	66	7.4

*S stands for the mobility measured by the space charge limited current (SCLC) method and O for the organic field effect transistor (OFET)method; N stands for the neat film and B for the blended film*.

In general, the three-dimensional (3D) or quasi-3D PDI derivatives have good compatibility with polymer donors and 3D charge-transporting channel. A star-shaped PDI acceptor (S(TPA-PDI)) with a triphenylamine (TPA) core displayed weak molecular aggregation and strong absorption as well as matched energy levels with PBDTTT-C-T. A PCE of 3.22% was achieved with 5% 1,8-diiodooctane (DIO) solvent additive (Lin et al., 2014b). A pyrene-fused PDI derivative (TPAPPDI) possessed upshifting LUMO energy level and low bandgap. The devices exhibited a PCE of 5.10% with ultra-high *V*_oc_ of up to 1.21V (Zhan et al., 2017). Two twisted propeller configuration PDI derivatives (TPH and TPH-Se) were developed. The investigations indicated that TPH-Se possessed more compact 3D network assembly due to the Se…O interactions. The PDBT-T1: TPH-Se solar cell showed a relatively high PCE of 9.28% while 8.28% for TPH based polymer solar cells (PSCs) (Meng et al., 2016a). Three PDI tetramers (TPC-PDI_4_, TPE-PDI_4_, and TPPz-PDI_4_) with twisted 3D structure were systematically studied through the relationship between structure and performance of 3D acceptor. The results revealed that intramolecular twist angle changed as the sequence of TPPz-PDI_4_ < TPE-PDI_4_ < TPC-PDI_4_. Although TPPz-PDI_4_ showed the strongest aggregation, it still had fine phase separation and effective charge transfer, and therefore, the highest PCE of 7.1% was obtained ascribed to high electron mobility (Lin et al., 2016a). Further transformed planar SF-PDI_2_ into a 3D molecular conformation created SF-PDI_4._ SF-PDI_4_ demonstrated a 3D interlocking geometry, which prevented excessive rotation and reinforcing conformational uniformity. The PCE of the PV4T2FBT: SF-PDI_4_ based devices was 5.98% (Lee et al., 2016). A PDI acceptor (TPB) exhibited cross-like molecular conformation but still partially conjugated with the BDTTh core. The PTB7-Th: TPB based solar cells achieved a PCE of 8.47% due to better conjugation and planarity (Wu et al., 2016). A star-shaped PDI derivative (PBI-Por) with porphyrin as central core was studied. Because porphyrin showed the large conjugated macrocycle and three characteristic absorption bands in the visible and NIR regions, the non-fullerene PSCs based on PBDB-T: PBI-Por blend films achieved a PCE of 7.4% (Zhang et al., 2017a).

## Acceptor-donor-acceptor (A-D-A) fused-ring electron acceptors

In recent years, A-D-A conjugated structures seem to be the most promising class of NFAs. The conjugated push-pull structure containing electron-rich and electron-poor units induces strong intramolecular charge transfer, which is beneficial to reducing the optical band gap. Moreover, variation of the donor or acceptor units can be used to regulate the HOMO or LUMO energy levels. The fused ring backbone facilitates electron delocalization and broadens absorption, and it can prevent the torsion or conformational transition of the molecular skeleton and enhance carrier mobility. The presence of the side chains on the conjugated backbone firstly ensures solution processing, in addition, and reduces molecular stacking, inhibits strong self-assembly and large phase separations.

### Fused tricyclic small molecule acceptors

**Figure 4:**
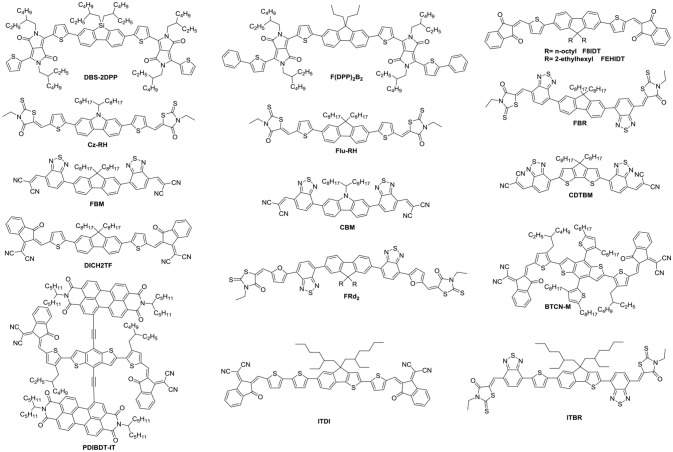
Chemical structures of fused tricyclic small molecule acceptors.

The three-membered ring is fused ring structure with the smallest size (Figure 4 and Table 3). Dibenzosilole (DBS) unit was the firstly to be used in A-D-A type NFAs, due to good electron-transporting properties, in addition to low-lying LUMO energy levels of silole moiety deriving from effective interactions between σ^*^-orbital of the silicon-carbon bond and π^*^-orbital of the butadiene. Diketopyrrolopyrrole (DPP) exhibits excellent light absorption and strong electron-withdrawing properties. A novel linear NFA (DBS-2DPP) based on DBS as central core and DPP as end group was reported in 2013, which possesses strong and broad absorption and moderate electron mobility. When P3HT was used as donor, the blended film formed fibrous nano-interpenetrating network, leading to a PCE of 2.05% (Lin et al., 2013). Based on this strategy, another DPP derivative (F(DPP)_2_B_2_) was developed. Consisting of fluorene as the core and two benzene end-capped DPP as the terminal, F(DPP)_2_B_2_ possessed excellent light-harvesting capability, moderate energy levels and good charge-transporting with the value of 2.8 × 10^−4^ cm^2^·V^−1^·s^−1^. While P3HT was also used as a donor material, the devices delivered a PCE of 3.1% with an extremely high *V*_oc_ (Shi et al., 2015). Because of concise synthesis and ready availability of fluorene, two isomeric acceptors (F8IDT and FEHIDT) using 2,3-dihydro-1*H*-indene-1,3-dione (ID) as end group were synthesized. The density functional theory calculations have shown that the LUMO energy of FxIDT were similar to that of fullerene derivatives, demonstrating FxIDT can be potentially used as acceptor materials. The devices based on P3HT: FxIDT blend films showed different performance (1.67% for F8IDT; 2.43% for FEHIDT). The main reason can be attributed to the difference of LUMO energy levels and the degree of electronic coupling between molecules, leading to various and low *V*_oc_ (Winzenberg et al., 2013). To reach a higher *V*_oc_, 3-ethylrhodanine is a reasonable choice as end group relative to ID due to weaker electron-withdrawing nature. Two rhodanine-based acceptors (Cz-RH and Flu-RH) were obtained. Cz-RH and Flu-RH possessed high-lying LUMO energy levels of −3.50 and −3.53 eV, respectively, compared to F8IDT, resulting in an excellent *V*_oc_ of 1.03 V. The devices exhibited a good photovoltaic performance with PCE of 3.08% for P3HT: Flu-RH and 2.56% for P3HT: Cz-RH. The difference was mostly attributed to the *J*_sc_, which originated from the maximum EQE intensity of 40% and a more efficient charge transfer from donor to acceptor in P3HT: Flu-RH blend films with higher photoluminescence (PL) quenching efficiencies of 86.7% (Kim et al., 2014). Subsequently, another acceptor FBR, bearing fluorene core and 3-ethylrhodanine end group but flanked by electron-deficient benzothiadiazole (BT) rather than thiophene spacer, was reported. BT as linker extends the conjugation and enhances charge transport. FBR exhibited a nonplanar 3D molecular structure, which is helpful to suppressing large aggregation and achieve efficient exciton separation confirmed by PL quenching efficiencies of over 90%. When blended with P3HT, the device showed a PCE of 4.11% with high *V*_oc_ of 0.82 V as a result of high LUMO energy level compared to PC_60_BM. It is a pity that the *J*_sc_ and FF of P3HT: FBR is inferior to those of P3HT: PC_60_BM, which can be caused by the difference of devices thickness and faster geminate recombination. Moreover, large overlapping absorption in P3HT: FBR blend films limited the more photocurrent generation. To harvest more photons across the solar spectrum, a low bandgap polymer PffBT4T-2DT was used to replace wide bandgap P3HT as donor, the device achieved PCE up to 7.8% with improved *J*_sc_. The increase of *V*_oc_ is originated from deep HOMO energy levels of donor (Holliday et al., 2015; Baran et al., 2016). Except for the optimization of donor materials, modification of acceptor materials also play an important role in improving the light absorption properties. FRd_2_ was developed based on FBR, but incorporation of furan spacer between BT and rhodanine end group, which help extending π-conjugation and reducing optical ban gaps. Employing PTB7-Th as donor, the device exhibited a PCE of 9.4% with *J*_sc_ of 15.7 mA cm^−2^, which is the reported highest performance for fluorene-based acceptors so far (Suman et al., 2017). Dicyanovinyl (DCV) unit was also an excellent electron-accepting motif to build A-D-A NFAs, because target molecules containing DCV can induce intramolecular charge transfer and promote planarity, which tends to achieve improved carrier mobilities. A set of acceptors (FBM, CBM and CDTBM) flanked by BT as spacer and DCV as end group were systematic studied. FBM and CBM possessed similar electronic properties, but CDTBM exhibited res-shifted absorption and deep LUMO level. Thus, CDTBM obtained higher *J*_sc_ and FF but lower *V*_oc_, leading to similar performance with PCE of ~5% (Wang et al., 2016). Another stronger electron-withdrawing end-capping group, 2-(6-oxo-5,6-dihydro-4*H*-cyclopenta[c]thiophen-4-ylidene)malononitrile (IC), built upon the structure of DC, could lower the band gap of the acceptor. A easily synthesized and high yield acceptor DICTF, bearing fluorene central block and thiophene spacers as well as IC terminal group, was reported in 2016. DICTF has strong and complementary absorption in the visible region and matched energy levels with PTB7-Th. The devices delivered a PCE near 8% (Li et al., 2016a). Benzo[1,2-*b*:4,5-*b*′]dithiophene (BDT) and its derivatives as electron-rich units in conjugated polymers have been well studied and have demonstrated outstanding results. Recently, BTCN-M, in which 4,8-bis-thiophene-substituted benzo[1,2-*b*:4,5-*b*′]dithiophene (BDT-T) as central block and IC as end group linking with BDT-T by thiophene spacer, was synthesized. Due to high steric hindrance caused by alkyl side groups in the BDT unit, BTCN-M showed weak π-π stacking, tending to act as acceptor material. Therefore, the devices based on BTCN-M: PBDB-T blended films exhibited a outperforming PCE of 5.89% whereas only 0.29% for that of BTCN-M: PC_71_BM blended films (Liu et al., 2018b). Cross-conjugated small molecular acceptor PDIBDT-IT, which combined the advantages of both the A-D-A and PDI type acceptors, exhibited broad absorption band ranging from 300 to 700 nm. The devices based on PDIBDT-IT: PTB7-Th blended films exhibited a PCE of 6.06% (Liu et al., 2018e). It is worth noting that these electron-rich central core were based on symmetrical units, however, NFAs based on asymmetrical cores were promising acceptor materials. Such as, ITDI and ITBR incorporating indenothiophene as core delivered PCEs of 8.00 and 7.49% when blended with PBDB-T and PTB7-Th, respectively (Kang et al., 2017; Tang et al., 2017). Although this type of fused-ring acceptor has made some progress, most of acceptors exhibited wide or medium band gaps absorption with poor spectral coverage and encountered sub-optimal morphologies, therefore, relatively low *J*_sc_ and FF were obtained.

**Table 3:** Summary of the photophysical and photovoltaic properties of fused tricyclic small molecule acceptors.

**Acceptor**	**Donor**	***E*_g_ (eV)**	**LUMO/HOMO (eV)**	**μ_e_ (cm^2^·V^−1^·s^−1^)**	***V*_oc_ (V)**	***J*_sc_ (mA cm^−2^)**	**FF (%)**	**PCE (%)**
DBS-2DPP	P3HT	1.83	−3.28/−5.30	3.3 × 10^−4^(S,B)	0.97	4.91	43	2.05
F(DPP)_2_B_2_	P3HT	1.82	−3.39/−5.21	2.8 × 10^−4^(S,B)	1.18	5.35	50	3.17
F8IDT	P3HT	2.10	−3.75/−5.85	–	0.72	4.82	48	1.67
FEHIDT	P3HT	2.00	−3.95/−5.95	–	0.95	3.82	67	2.43
Cz-RH	P3HT	2.05	−3.50/−5.53	–	1.03	4.69	53	2.56
Flu-RH	P3HT	2.10	−3.53/−5.58	–	1.01.3	5.70	52	3.08
FBR	P3HT	2.14	−3.57/−5.70	2.6 × 10^−5^(S,B)	0.82	7.95	63	4.11
FBR	Pff4TBT-2DT	2.14	−3.75/−5.83	3.8 × 10^−4^(S,B)	1.12	11.5	61	7.80
FRd_2_	PTB7-Th	2.09	−3.58/−5.67	4.3 × 10^−4^(S,B)	0.83	15.7	72	9.40
FBM	PTB7-Th	2.11	−3.67/−6.18	1.0 × 10^−6^(S,B)	0.88	11.2	51	5.10
CBM	PTB7-Th	2.02	−3.64/−6.10	1.9 × 10^−6^(S,B)	0.88	10.6	53	5.30
CDTBM	PTB7-Th	1.45	−3.90/−5.79	1.8 × 10^−6^(S,B)	0.66	11.9	60	5.00
DICTF	PTB7-Th	1.88	−3.79/−5.67	5.85 × 10^−5^(S,B)	0.86	16.6	56	7.93
BTCN-M	PBDB-T	1.63	−3.95/−5.69	2.91 × 10^−5^(S,N)	0.98	12.3	50	5.89
PDIBDT-IT	PTB7-Th	1.69	−3.97/−5.95	3.51 × 10^−5^(S,B)	0.74	13.6	61	6.06
ITDI	PBDB-T	1.53	−4.18/−5.89	9.15 × 10^−6^(S,N)	0.94	14.23	60	8.00
ITBR	PTB7-Th	1.71	−3.71/−5.55	1.51 × 10^−5^(S,B)	1.02	14.46	51	7.49

S stands for the mobility measured by the space charge limited current (SCLC) method and O for the organic field effect transistor (OFET)method; N stands for the neat film and B for the blended film

### Fused pentacyclic small molecule acceptors

**Figure 5:**
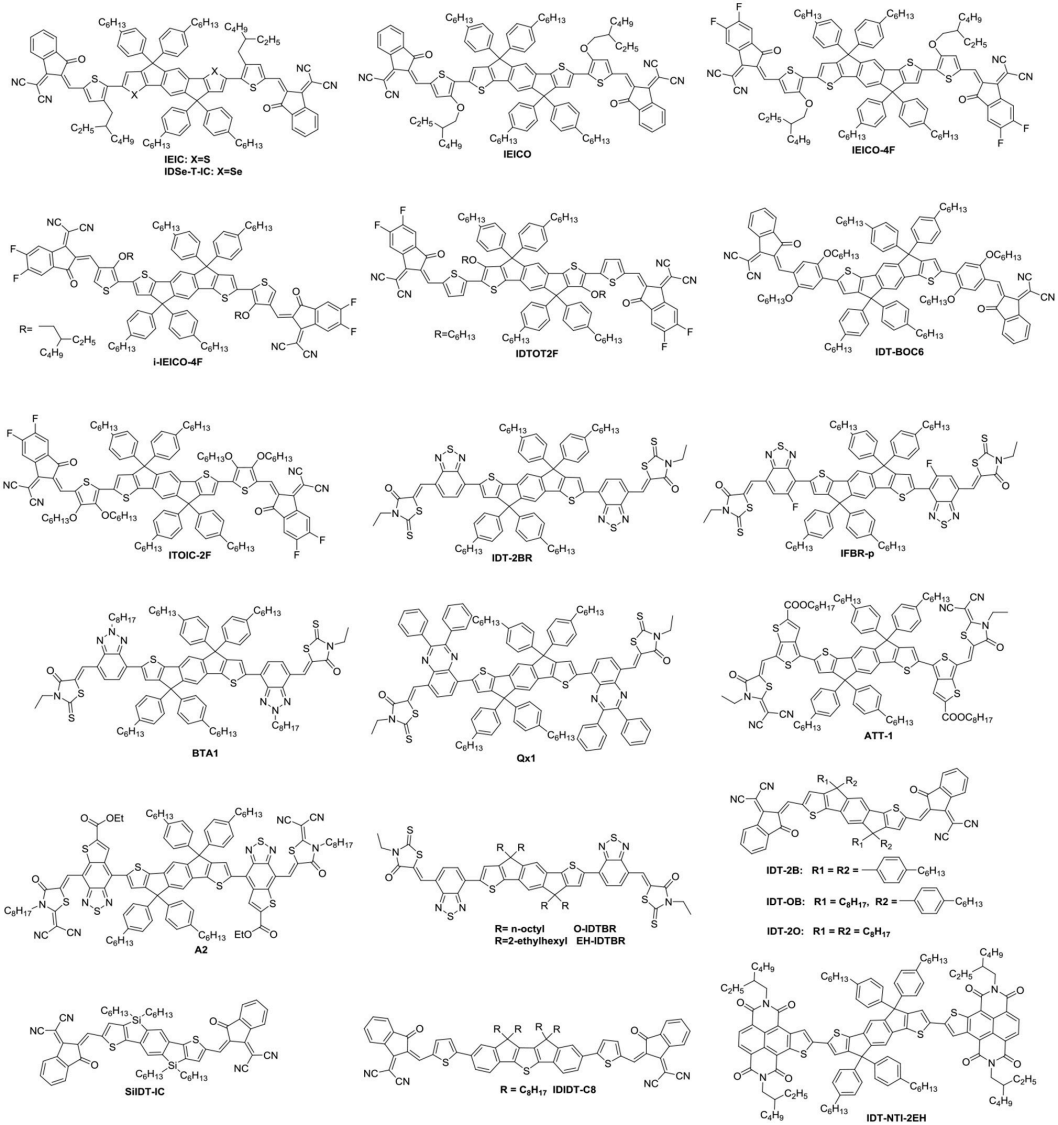
Chemical structures of fused pentacyclic small molecule acceptors.

Indacenodithiophene (IDT) is the most representative fused pentacyclic donor unit in A-D-A acceptors due to rigid and coplanar structure for good absorption and excellent charge mobility. Moreover, the side chain substituents of the conjugated block can ensure the solution processability and inhibit strong self-assembly of molecules (Figure 5 and Table 4).

**Table 4:** Summary of the photophysical and photovoltaic properties of fused pentacyclic small molecule acceptors.

**Acceptor**	**Donor**	***E*_g_ (eV)**	**LUMO/HOMO (eV)**	**μ_e_ (cm^2^·V^−1^·s^−1^)**	***V*_oc_ (V)**	***J*_sc_ (mA cm^−2^)**	**FF (%)**	**PCE (%)**
IEIC	PTB7-Th	1.57	−3.82/−5.42	1.00 × 10^−4^(S,B)	0.97	13.55	48	6.31
IEIC	PffT2-FTAZ-2DT	1.57	−3.88/−5.45	2.10 × 10^−4^(S,B)	1.00	12.70	62	7.30
IDSe-T-IC	J51	1.52	−3.79/−5.45	7.72 × 10^−5^(S,B)	0.91	15.20	62	8.58
IEICO	PBDTTT-E-T	1.34	−3.95/−5.32	1.50 × 10^−3^(S,B)	0.82	17.70	58	8.40
IEICO-4F	PTB7-Th	1.29	−4.19/−5.44	1.48 × 10^−4^(S,B)	0.71	27.3	66	12.1
i-IEICO-4F	J52	1.56	−3.33/−5.01	3.83 × 10^−4^(S,B)	0.85	22.86	68	13.18
IDTOT-2F	PBDB-T	1.44	−3.94/−5.54	4.99 × 10^−4^(S,B)	0.85	20.87	72	12.79
IDT-BOC6	PBDB-T	1.63	−3.78/−5.51	4.00 × 10^−5^(S,B)	1.01	17.52	54	9.60
ITOIC-2F	PBDB-T	1.45	−3.87/−5.57	6.02 × 10^−4^(S,B)	0.90	21.04	65	12.17
IDT-2BR	P3HT	1.68	−3.69/−5.52	2.60 × 10^−4^(S,B)	0.84	8.91	68	5.12
IFBR-p	PTzBI	1.67	−3.73/−5.64	1.50 × 10^−4^(S,B)	1.00	11.9	63	7.44
BTA1	P3HT	1.85	−3.55/−5.51	3.20 × 10^−5^(S,B)	1.02	9.93	57	5.24
Qx1	P3HT	1.74	−3.60/−5.42	3.20 × 10^−5^(S,B)	1.00	6.02	67	4.03
ATT-1	PTB7-Th	1.54	−3.63/−5.50	2.40 × 10^−4^(S,B)	0.87	16.48	70	10.07
A2	PTB7-Th	1.36	−3.78/−5.70	2.30 × 10^−4^(S,B)	0.71	20.33	63	9.07
O-IDTBR	P3HT	1.63	−3.88/−5.51	4.70 × 10^−6^(S,B)	0.73	14.10	63	6.40
EH-IDTBR	P3HT	1.68	−3.90/−5.58	6.10 × 10^−6^(S,B)	0.77	12.2	64	6.05
IDT-2B	PBDB-T	1.73	−3.84/−5.80	1.26 × 10^−5^(S,B)	0.89	13.30	54	6.42
IDT-OB	PBDB-T	1.66	−3.87/−5.77	2.71 × 10^−4^(S,B)	0.88	16.18	71	10.12
IDT-2O	PBDB-T	1.64	−3.85/−5.73	7.12 × 10^−5^(S,B)	0.86	15.64	72	9.68
SiIDT-IC	PBDB-T	1.69	−3.78/−5.47	1.02 × 10^−4^(S,B)	0.92	13.53	66	8.16
IDIDT-C8	PBDB-T	1.63	−3.86/−5.50	2.41 × 10^−5^(S,B)	0.97	15.81	66	10.10
IDT-NTI-2EH	PBDB-T	1.59	−3.90/−5.40	1.40 × 10^−3^(S,B)	0.92	14.48	69	9.07

*S stands for the mobility measured by the space charge limited current (SCLC) method and O for the organic field effect transistor (OFET)method; N stands for the neat film and B for the blended film*.

**Scheme 2:**
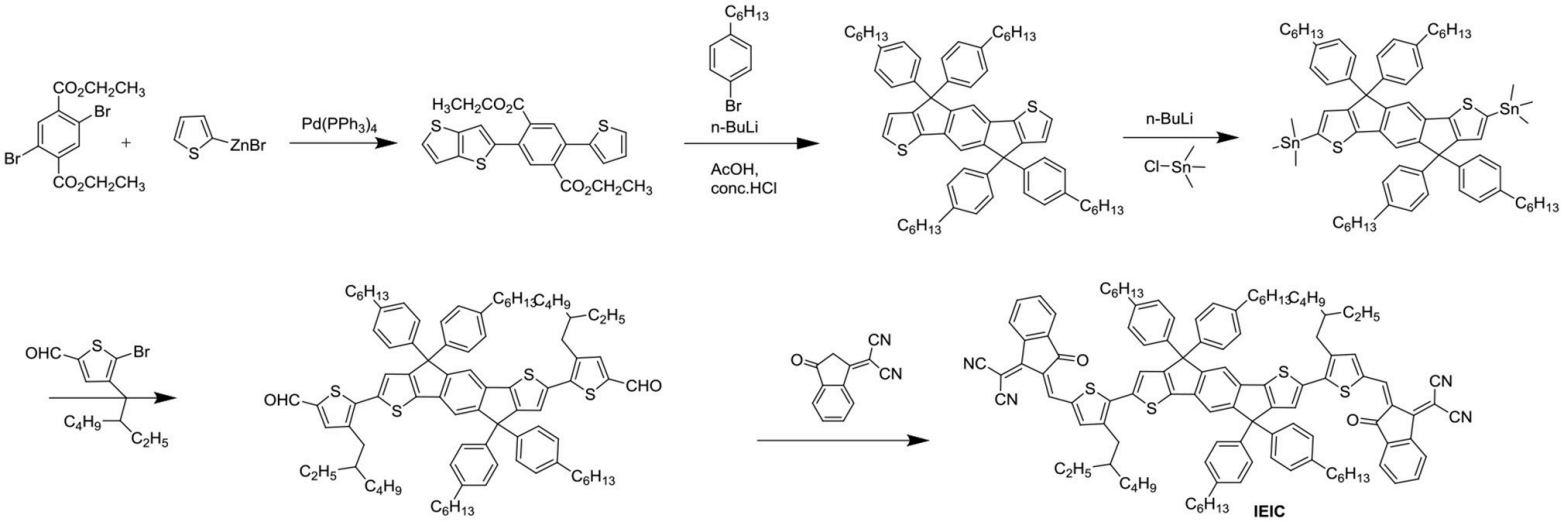
Synthetic route of IEIC.

IEIC with IDT as the core flanked by thiophene spacers and IC end groups was studied (Scheme 2). IEIC showed strong absorption in the 500–750 nm region with an extinction coefficient of 1.1 × 10^5^ M^−1^ cm^−1^ at 672 nm and relatively high electron mobility of 2.1 × 10^−4^ cm^2^ V^−1^ S^−1^. The blend films of PTB7-Th as donor and IEIC as acceptor showed nanoscale interpenetrating morphology, thereby a PCE of 6.31% was achieved (Lin et al., 2015c). The limitation of the PCE was poor FF and *J*_sc_, which mainly came from the big overlapped absorption profiles and imbalanced charge mobility of active layer. With this in mind, when IEIC was laterly blended with a large bandgap donor polymer PffT2-FTAZ-2DT, the PCE reached 7.30%. The improved PCE can be attributed to complementary absorption and balanced charge mobility as well as moderate phase domain size (Lin et al., 2015a). IEIC was the first acceptor material using IDT as central core, and exhibited good performance at that time, providing a good theoretical basis for the later fused pentacyclic small molecule acceptors. The synthetic steps of ITIC were shown in Scheme 2. Using larger and looser outermost electron cloud, selenium atoms to replace sulfur atoms afford IDSe-T-IC, which possessed decreased bandgap of 1.52 eV and improved LUMO energy level as well as increased carrier mobility. Thus a high PCE of 8.58% was obtained with a large bandgap polymer J51 as donor (Li et al., 2016d). Designing and synthesizing low bandgap acceptor materials can also make better use of solar spectrum to absorb larger fractions of photons. IEICO, replacing alkyl groups with alkoxy groups, was reported with *E*_g_ of 1.34 eV. Introduction of alkoxyl chains increased the HOMO energy level but had little effect on the LUMO level. By employing PBDTTT-E-T as the donor, the IEICO-based devices delivered a high PCE of 8.4% with an increased *J*_sc_ of 17.70 mA cm^−2^ (Yao et al., 2016). A further development of IEICO obtained IEICO-4F by introducing F atoms in the IC end groups. When blended with a narrow bandgap polymer PTB7-Th, a PCE of 12.8% was achieved (Wang et al., 2018a). i-IEICO-4F, an isomer of IEICO-4F by attaching the end groups in the 4-position instead of 5-position at the neighboring spacers, is a twisted configuration, resulting in blue shifts and complementary absorption with the wide-bandgap polymer J52. The devices based on i-IEICO-4F delivered an excellent PCE of 13.18% (Song et al., 2018). Introducing alkoxyl side chains at the central core rather than thiophene spacer provided IDTT2F, which exhibited excellent solubility and ordered molecular packing, resulting in a PCE of 12.79% blended with PBDB-T (Liu et al., 2018d). IDT-BOC6 was also synthesized by using IDT as central core and IC as end groups, but bis(alkoxy)-substituted benzene ring as spacer. Incorporation of alkoxyl groups not only increased the LUMO energy levels but also induced conformational control and enhanced the planarity. IDT-BOC6 locked by intramolecular noncovalent interactions displayed a broad absorption spectrum, high electron mobility and weak nonradiative recombination. The devices based IDT-BOC6 afforded a PCE of 9.6% with PBDB-T as donor (Liu et al., 2017b). ITOIC-2F was also included noncovalently conformational locking, the corresponding devices delivered a PCE of 12.17% when blended with PBDB-T (Liu et al., 2018f).

A planar electron acceptor IDT-2BR was synthesized, which IDT was as core flanked by BT as the first electron-withdrawing group and the second electron-deficient 3-ethylrhodanine units on the periphery. The P3HT: IDT-2BR blended films exhibited clear interpenetrating networks and moderate phase separation with the addition of 3% CN. The devices achieved a PCE of 5.12% with a high FF of 68% due to balanced charge mobilities (Wu et al., 2015). IFBR-p was synthesized by incorporating fluorine atoms on the BT unit of IBT-2BR.The OSCs based on PTzBI: IFBR-p blend film showed a PCE as high as 7.44% as result of intermolecular and intramolecular interactions induced by C–H…F non-covalent force (Zhong et al., 2017). BTA1, containing benzo[d][1,2,3]triazole (BTA) as spacer, is analogous to the IBT-2BR acceptor. BTA was a weaker electron-deficient unit than BT, which would make it possess a higher LUMO energy level. Also while blended with P3HT, the device gives the PCE of 5.24% with a high *V*_oc_ of 1.02 V (Xiao et al., 2017a). Meanwhile, quinoxaline (Qx) is the other weak electron-drawing unit and has been copolymerized with different electron-rich building block to get high performance D-A polymers (Yuan et al., 2017). Qx1, using Qx as bridge, was synthesized and explored. The devices based on P3HT: Qx1 blend films achieved a PCE of 4.03% with a *V*_oc_ of 1.00 V (Xiao et al., 2018). ATT-1 can be considered as a IDT-2BR derivative, which used ester-substituted thieno[3,4-b]thiophene as spacer and 2-(1,1-dicyanomethylene) rhodanine as end group. ATT-1, adopting quinoidal resonance to extend the π-conjugation and enhance the absorption, exhibits a broad absorption with a high absorption coefficient of 1.2 × 10^5^ L mol^−1^ cm^−1^and slightly high LUMO energy level. When blended with PTB7-Th, the devices achieved a PCE of 10.07% after the addition of DIO. It's worthy to note that the PCE was only 4.46% without any post-treatment,. The investigations indicated that the addition of DIO provided an ideal morphology for efficient charge transport (Liu et al., 2016a). The design of ATT-1 was further developed by substituting the thieno[3,4-b]thiophene spacer with thiophene-fused benzothiadiazole (BTT) unit as p-bridge to obtain A2. The BTT unit connecting on the IDT core not only extend the conjugation length, but also stabilize the quinoid conjugation system, which resulted in red-shift absorption and low bandgap of 1.36 eV. Ultimately, the PCE of 9.07% was reached with an excellent *J*_sc_ of 20.33 mA cm^−2^ (Xu et al., 2018a). IDT-BR with IDT core was designed and synthesized to solve the issues of FBR, including the large spectra overlap and poor charge percolation pathway. IDTBR had significantly red-shift absorption and tended to crystallize on length scales, meanwhile, O-IDTBR with linear alkyl chains was a more crystalline acceptor and had a further red-shift absorption. The resulting OSCs based on O-IDTBR achieved a PCE of 6.4% while 6.05% for EH-IDTBR (Holliday et al., 2016). Alkyl and alkylaryl groups have been widely used as side chains of IDT to ensure solubility and suppress strong aggregations. Compared with alkylaryl units, alkyl substituents enable π-π stacking. However, alkyl substituted acceptor usually formed large domains, leading to incomplete exciton separation. Thus a new acceptor IDT-OB with asymmetric side chains was reported, which reduced strong self-assembly but still had close packing in film due to the existence of more configurationally isomers. As a result, 10.12% was reached for IDT-OB based devices without any post-treatment, while the PCE of 9.68% for IDT-2O, the performance based on IDT-2B was the worst, with only 6.42% efficiency (Feng et al., 2017c). By the replacement of the C-bridge of IDT with the Si-bridge, SiIDT-IC was obtained. Introduction of Si atom can result in a high-lying LUMO energy level to achieve a high *V*_oc_. When blended with PBDB-T, the devices delivered a PCE of 8.16% with high *V*_oc_ of 0.92 V, but the performance is lower than the corresponding C-bridge acceptor (8.83%) due to inferior *J*_sc_ (Nian et al., 2018). Most of the reported NFAs are trans-arranged side chains linked with the central core. However, IDIDT-C8 with cis-arranged alkyl side chains had weaker π-π stacking than that of trans-arranged one. The blend films with PBDB-T as donor exhibited moderate molecular packing and film morphology, especially, IDIDT-C8 showed a good crystallinity and face-on orientation, resulting in an excellent PCE of 10.10% (Hou et al., 2018). Naphthalene diimide (NDI) was broadly used as acceptor unit due to their strong electron affinity and excellent electron transport properties. Naphtho[2,3-*b*]thiophene diimide (NTI) was connected on the IDT core to give a new acceptor IDT-NTI-2EH, which had red-shifted absorption and strong π-π stacking due to planar conjugated structure. The corresponding devices showed a PCE of 9.07% with PBDB-T as donor (Hamonnet et al., 2017).

### Fused heptacyclic small molecule acceptors

**Figure 6:**
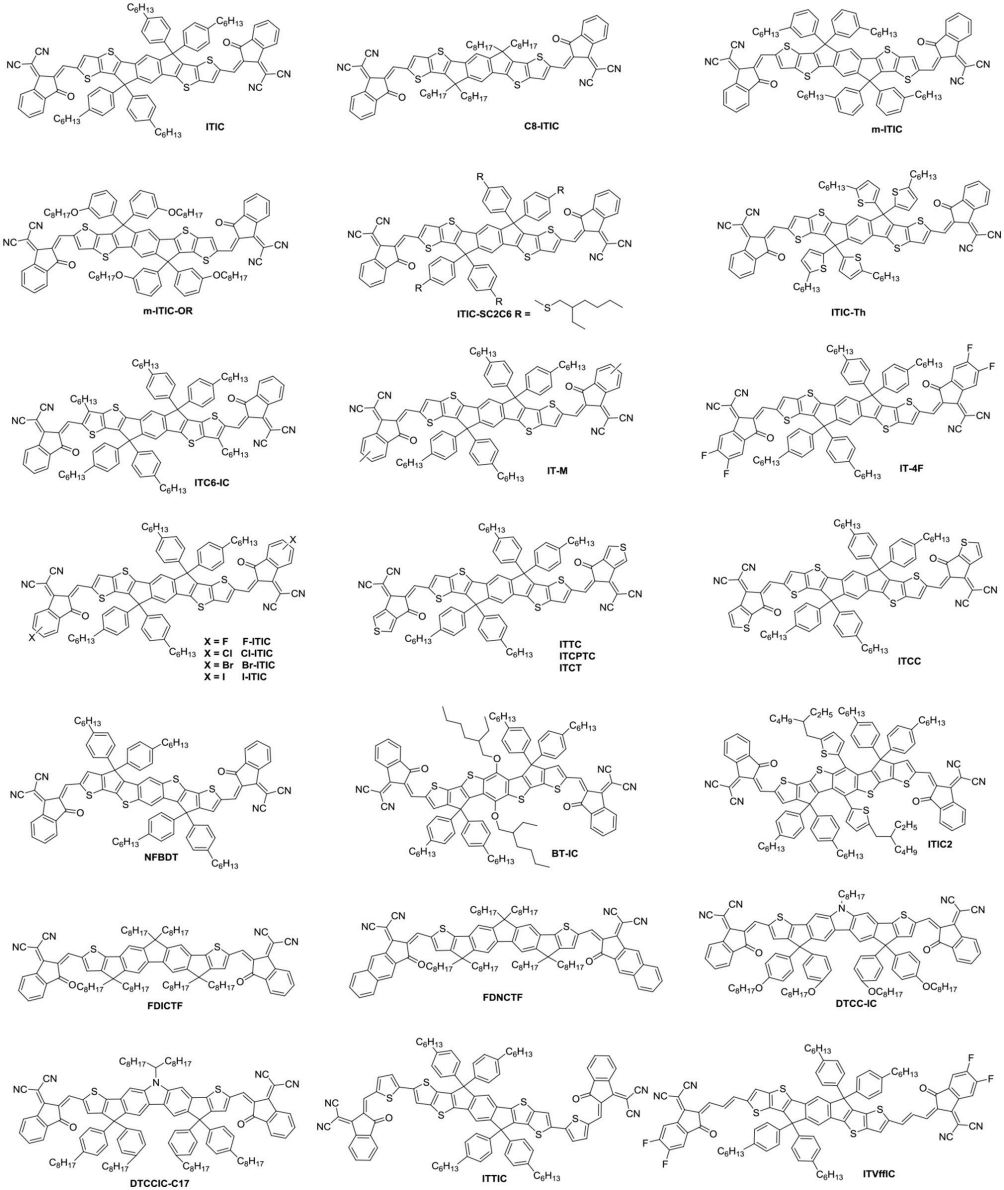
Chemical structures of fused heptacyclic small molecule acceptors.

**Scheme 3:**
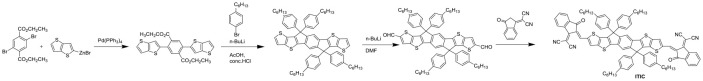
Synthetic route of ITIC.

Indacenodithieno[3,2-*b*]thiophene (IDTT) was a further development of the IDT, from a fused pentacyclic to a fused heptacyclic structure (Figure 6 and Table 5). The first A-D-A acceptor based on IDTT was ITIC, which IDTT was used as core directly flanked by IC. ITIC possessed strong and broad absorption in the visible and even NIR region, matched energy levels and good miscibility with PTB7-Th. The resulting OSCs based on PTB7-Th: ITIC blend films exhibited a promising PCE of 6.8%, which was better than that of the devices based on PTB7-Th: PC_61_BM (Lin et al., 2015b). Since then, the ITIC-based OSCs have shown high photovoltaic performance with multiple polymer donors (Bin et al., 2016; Xia et al., 2016; Yuan et al., 2016; Yu et al., 2017; Hu et al., 2018; Liu et al., 2018a; Xu et al., 2018b). ITIC was the first and a successful fused heptacyclic small molecule acceptors. The synthetic steps of ITIC were shown in Scheme 3. After that, much effort has been devoted to the modifications of ITIC structure, for example, by manipulating the aromatic core and changing the side chains as well as substituting the electron deficient end-capping groups (Wei et al., 2017; Alamoudi et al., 2018; Yang et al., 2018b).

**Table 5:** Summary of the photophysical and photovoltaic properties of fused heptacyclic small molecule acceptors.

**Acceptor**	**Donor**	***E*_g_ (eV)**	**LUMO/HOMO (eV)**	**μ_e_ (cm^2^·V^−1^·s^−1^)**	***V*_oc_ (V)**	***J*_sc_ (mA cm^−2^)**	**FF (%)**	**PCE (%)**
ITIC	PTB7-Th	1.59	−3.83/−5.48	1.10 × 10^−4^(S,B)	0.81	14.21	59	6.80
C8-ITIC	PFBDB-T	1.53	−3.91/−5.63	–	0.94	19.6	72	13.2
m-ITIC	J61	1.58	−3.82/−5.52	2.45 × 10^−4^(S,N)	0.91	18.31	71	11.77
m-ITIC-OR	HFQx-T	1.65	−3.97/−5.65	2.02 × 10^−4^(S,B)	0.90	16.15	64	9.30
ITIC-SC2C6	PBDB-ST		−3.86/−5.74	5.43 × 10^−4^(S,B)	0.92	15.81	63	9.16
ITIC-Th	PTB7-Th	1.60	−3.93/−5.66	4.50 × 10^−4^(S,B)	0.80	15.93	68	8.7
ITIC-Th	PDBT-T1	1.60	−3.93/−5.66	4.20 × 10^−4^(S,B)	0.88	16.24	67	9.6
ITC6-IC	PBDB-T	1.60	−3.92/−5.73	–	0.97	16.41	73	11.61
IT-M	PBDB-T	1.60	−3.98/−5.58	1.10 × 10^−4^(S,B)	0.94	17.44	74	12.05
IT-4F	PBDB-T-SF	1.51	−4.14/−5.66	4.20 × 10^−4^(S,B)	0.88	20.88	71	13.10
F-ITIC	PTPDBDT	1.56	−4.09/−5.65	3.10 × 10^−4^(S,B)	0.94	14.1	66	8.8
Cl-ITIC	PTPDBDT	1.56	−4.14/−5.70	5.20 × 10^−4^(S,B)	0.94	15.6	65	9.5
Br-ITIC	PTPDBDT	1.53	−4.20/−5.73	5.10 × 10^−4^(S,B)	0.93	15.4	66	9.4
I-ITIC	PTPDBDT	1.55	−4.14/−5.68	4.10 × 10^−4^(S,B)	0.95	14.5	65	8.9
ITTC	HFQx-T	1.61	−3.85/−5.49	1.44 × 10^−4^(S,B)	0.88	16.49	71	10.4
ITCPTC	PBT1-EH	1.58	−3.96/−5.62	2.69 × 10^−3^(S,B)	0.95	16.5	75	11.8
ITCT	PBDB-T	1.59	−4.02/−5.66	5.10 × 10^−4^(S,B)	0.86	18.1	73	11.27
ITCC	PBDB-T	1.67	3.76/−5.47	6.74 × 10^−4^(S,B)	1.01	15.9	71	11.4
NFBDT	PBDB-T	1.56	−3.83/−5.40	1.38 × 10^−4^(S,B)	0.87	17.85	67	10.42
BT-IC	J71	1.43	−3.85/−5.32	3.53 × 10^−4^(S,B)	0.90	17.75	66	10.46
ITIC2	FTAZ	1.53	−3.80/−5.43	4.10 × 10^−4^(S,B)	0.93	18.88	63	11.0
FDICTF	PBDB-T	1.63	−3.71/−5.43	3.79 × 10^−5^(S,N)	0.95	16.0	67	10.0
FDNCTF	PBDB-T	1.60	−3.73/−5.42	2.83 × 10^−4^(S,N)	0.94	16.5	73	11.2
DTCC-IC	PTB7-Th	1.59	−3.87/−5.50	1.86 × 10^−3^(S,B)	0.95	11.23	56	6.0
DTCCIC-C17	PBDB-T	1.60	−3.65/−5.46	-	0.97	14.27	68	9.48
ITTIC	PBDB-T1	1.46	−3.82/−5.28	1.08 × 10^−4^(S,B)	0.92	15.93	62	9.12
ITVffIC	J71	1.35	−4.04/−5.58	2.15 × 10^−4^(S,B)	0.81	2.60	63	10.54

*S stands for the mobility measured by the space charge limited current (SCLC) method and O for the organic field effect transistor (OFET)method; N stands for the neat film and B for the blended film*.

It is well-established that the length, type and branch position of side chains play an important role in electronic properties and intermolecular self-assembly. C8-ITIC with four linear octyl side chains was reported for comparison with ITIC. C8-ITIC possessed a lower optical band gap, higher absorption coefficient and increased crystallinity. Blending with PFBDB-T, the devices delivered a PCE up to 13.2% while the devices based on ITIC showed only 11.71% efficiency (Fei et al., 2018). A new acceptor m-ITIC with meta-alkyl-phenyl side groups was synthesized to investigate the effects of side-chain isomerism. This work showed that m-ITIC had higher absorption coefficient, more crystallinity, and increased electron mobilities in comparison with ITIC. The resulting OSCs based on m-ITIC demonstrated a higher PCE of 11.77% than 10.57% for ITIC with a medium bandgap polymer J61 as donor (Yang et al., 2016). Actually, alkoxyphenyl side chains seemed more easily synthesized *via* simple etherification, which is beneficial for large scale production. The m-ITIC-OR bearing IDTT core with meta-alkoxyphenyl side chains and IC as end groups was reported. The HFQx-T: m-ITIC-OR blend films possessed high and balanced charge transport, negligible bimolecular recombination resulting in a promising PCE of 9.3%, which was higher than 9.07% efficiency of ITIC based devices under the same conditions (Zhang et al., 2017c). ITIC-SC2C6 based on branched 4-(alkylthio)-phenyl side chains was systematically explored. The investigations indicated that this acceptor had improved solubility, which is helpful for polymer donor to form nanofibrils. Consequently, the OSCs exhibited a PCE of 9.16% with PBDB-ST as donor (Zhang et al., 2017b). ITIC-Th, replacing phenyl side chains of ITIC with thienyl side groups, exhibited low LUMO energy levels, which can match with low bandgap and wide bandgap polymer donor. Additionally, ITIC-Th possessed high electron mobility owing to enhanced intermolecular interactions induced by S…S interaction. The OSCs were fabricated by blending ITIC-Th with low bandgap polymer PTB7-Th and wide bandgap polymer PBDB-T1, the PCE reached 8.7 and 9.6%, respectively (Lin et al., 2016b). ITIC and its derivatives has inevitable steric isomers between donor units and end groups linking by C = C covalent bond. To solve this defect, a definite molecular conformation ITC6-IC, which long alkyl chains were introduced into the terminal of IDTT, was synthesized and discussed. ITC6-IC exhibited planar structure, good solubility, high-lying LUMO energy levels and enhanced compatibility with donor materials. The blend films with PBDB-T as polymer donor and ITC6-IC as acceptor showed a fibril crystallization with bicontinuous network morphology after thermal annealing. Consequently, the OSCs revealed a promising PCE of 11.61% with a high *V*_oc_ of 0.97 V (Zhang et al., 2018d).

Density functional theory calculations reveal that the LUMO mainly delocalizes at the end groups of the ITIC derivatives. Furthermore, the side chains on the central donor units would hinder the tight stacking of the molecules, thus, the stacking of the end groups are likely to provide the main electron transport pathway. Indeed, atomistic molecular dynamic simulations referred that local intermolecular π-π stacking between the acceptor units of the ITIC film led to 3D molecular packing (Han et al., 2017; Yan et al., 2018). As a consequence, the reasonable regulation of ITIC terminal units is excepted to obtain higher LUMO energy level and better isotropic electron transport characteristics. A methyl group was introduced onto the phenyl of the IC to give IT-M, which elevates the LUMO energy levels due to the weak electron-rich properties of methyl. The devices demonstrated a high PCE of 12.05% with *V*_oc_ of 0.94 V when blended with PBDB-T donor, which is the highest value for single-junction OSCs at that time (Li et al., 2016c). Then, replacing the methyl groups with the most electronegative fluorine atoms provided the new acceptor IT-4F. Although the fluorine atom resulted in low LUMO level, it had good crystallinity and high electron transport properties from noncovalent interactions of F…H,S…F and so on. The resulting OSCs based on PBDB-T-SF: IT-4F achieved a high PCE of 13.1% (Zhao et al., 2017). A similar effect of good crystallinities and noncovalent interactions can be found in other halogenated non-fullerene small molecular Cl-ITIC, Br-ITIC, and I-ITIC. The devices based on these halogenated acceptors showed PCEs of 9.5, 9.4, and 8.9%, respectively, which are higher than that of F-ITIC (8.8%) under the same circumstances (Yang et al., 2017). There are similar phenomena in other systems (Li et al., 2017c; Wang et al., 2018b). Apart from changing the substituents of the end groups, the modification of aromatic structure also attracted attention. Replacing the benzene of the IC with thiophene units gave isomers ITTC, ITCPTC, ITCT, and ITCC, which all show good potential in OSCs due to enhanced intermolecular π-π interaction induced by S…S interactions. When ITTC (or ITCPTC) as acceptor, the OSCs based on HFQx-T donor achieved a PCE of 10.4% (Zhang et al., 2017d) and 11.8% with PBT1-EH as donor (Xie et al., 2017). When PBDB-T as donor, the device based on ITCC delivered a PCE of 11.4% (Yao et al., 2017) and 11.27% for ITCT based devices (Liu et al., 2018c). Introducing methyl group onto the thiophene unit can also increase the *V*_oc_ (Cui et al., 2017; Luo et al., 2018).

NFBDT is an isomer of ITIC, which based on a heptacyclic benzodi(cyclopentadithiophene) (FBDT) unit as core and IC as end groups. Due to symmetric and planar conjugated structure of BDT unit, the NFBDT possessed low bandgap of 1.56 eV. When blended with PBDB-T, the devices showed a PCE of 10.42% (Kan et al., 2017). Introducing 2-ethylhexyloxy on the BDT unit obtained BT-IC to further reduce *E*_g_ (1.43 eV) by elevating the HOMO energy levels. The OSC fabricated by blending J71 and BT-IC achieved a PCE of 10.5% (Li et al., 2017d). ITIC2 with 5-(2-ethylhexyl) thiophene as side chains was a further development of NFBDT. The conjugated side chains is helpful for enhancing the absorption, intermolecular interaction and π-π stacking. Thus a high PCE of 11.0% was obtained while blended with FTAZ donor (Wang et al., 2017a). Fusing the thiophene spacers to the fluorene of DICTF afforded ladder acceptor FDICTF, leading to narrow bandgap, higher extinction coefficient and slightly higher LUMO energy level. In addition to extending the central core to enhanced absorption and intermolecular overlaps, extending conjugation end groups was also an effective strategy. For example, FDNCTF was obtained by replacing the benzene units of FDICTF with naphthalene units. The devices based on FDNCTF blended with PBDB-T delivered a higher PCE of 11.2% compared to 10.0% for FDICTF (Feng et al., 2017a; Qiu et al., 2017). Two acceptors DTCC-IC and DTCCIC-C17 based on dithienocyclopentacarbazole (DTCC) with different side chains were reported. Due to strong electron-donating properties and coplanar conjugated skeleton, both of them possessed strong absorption and good performance with PCE of 6.0% for DTCC-IC and 9.48% for DTCCIC-C17 (Cao et al., 2017; Hsiao et al., 2017). Two narrow bandgap acceptors ITTIC (1.46 eV) and ITVffIC (1.35 eV) were synthesized by incorporating thiophene units and double-bond as spacers, respectively. The PSCs based on PBDB-T1: ITTIC showed a PCE of 9.12% without any additives, and 10.54% for J71: ITVffIC blend films (Li et al., 2017b; Zhang et al., 2017e).

### Other fused-ring small molecule acceptors

**Figure 7:**
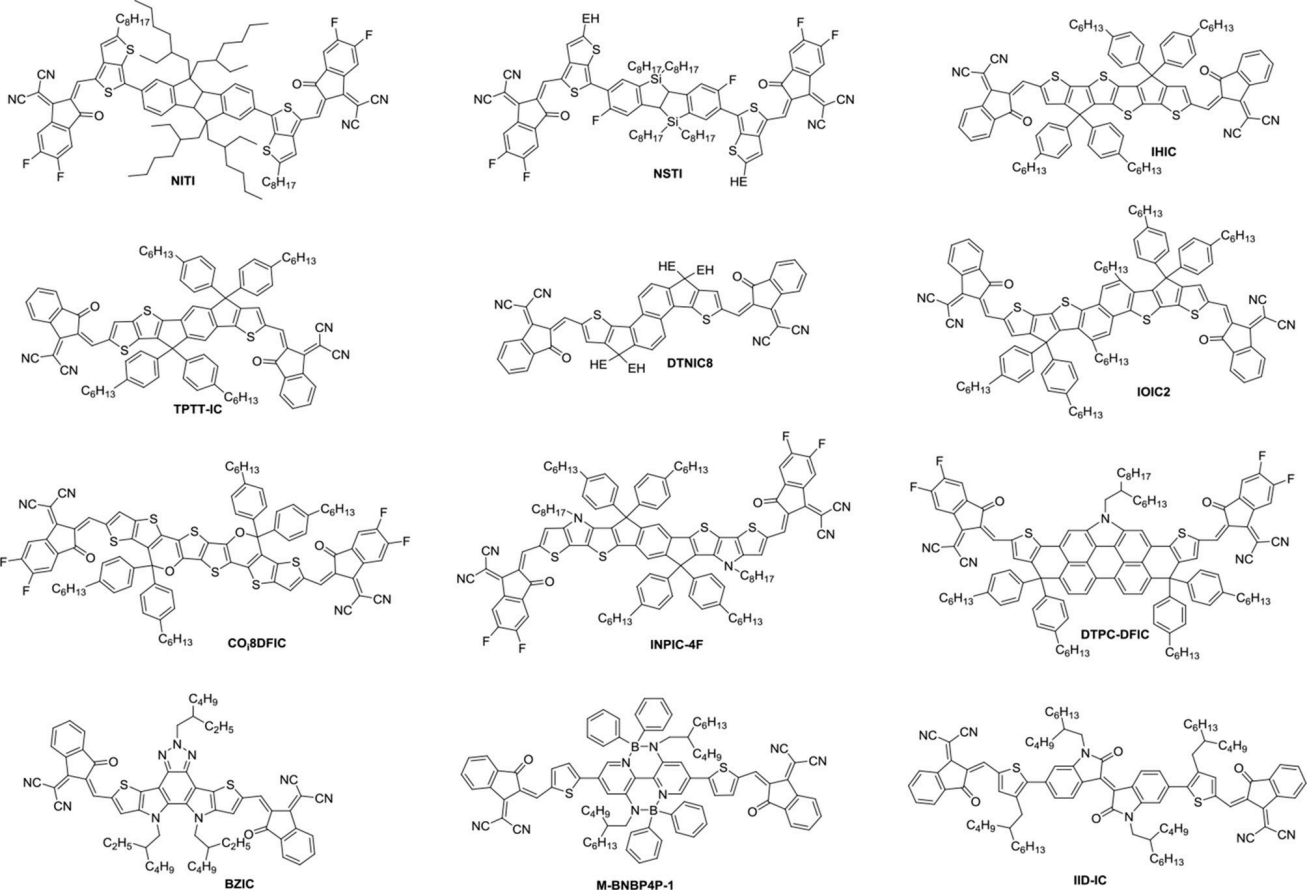
Chemical structures of other fused-ring small molecule acceptors.

NITI, bearing an indenoindene core which is a carbon-bridged E-stilbene with a centrosymmetry, exhibited a low optical bandgap of 1.49 eV and high extinction coefficient of 1.90 × 10^5^ cm^−1^ (Figure 7 and Table 6). The corresponding devices delivered an excellent PCE of 12.74% by blending a large bandgap polymer PBDB-T due to the good charge transport property and proper phase separation (Xu et al., 2017). A further development of NITI was by replacing carbon-bridge with silicon-bridge to give NSTI. Bis-silicon-bridged stilbene (BSS) has rigid and coplanar structure and four side chains can suppress the strong aggregations. When blended with PBDB-T, the OSCs obtained a PCE of 10.33% with CN as additive (Zhang and Zhu, 2018). IHIC was a fused hexacyclic small molecule acceptor and the central core consisted of thieno[3,2-*b*]thiophene ring and two terminal thiophene. The thiophene-rich cores possessed symmetrical, rigid and coplanar structure, IC was used as the end group to construct push-pull structure, which is beneficial to inducing ICT and shifting the absorption spectrum to the NIR region. The IHIC showed strong NIR absorption with a narrow bandgap of 1.38 eV and a high electron mobility of 2.4 × 10^−3^ cm^2^ V^−1^ s^−1^. The semitransparent OSCs achieved a PCE of 9.77% with a visible transmittance of 36% when blended with PTB7-Th (Wang et al., 2017b). Other similar thiophene-rich acceptors had strong NIR absorption and showed excellent performance (Jia et al., 2017; Dai et al., 2018; Li et al., 2018b). TPTT-IC was synthesized with a asymmetric thiophene-phenylene-thieno[3,2-*b*]thiophene-fused central core. Dipole-dipole interactions of asymmetric molecules tend to form strong π-π stacking on the face on orientation and achieved high FF (Li et al., 2018a). The OSCs achieved a PCE of 10.5% while blended with wide bandgap polymer PBT1-C (Li et al., 2018a). DTNIC8 can be considered as a further development of the IHIC by replacing thieno[3,2-*b*]thiophene with naphthalene. The angular-shaped central core dithienonaphthalene (DTN) had a more extended π-conjugation system in comparison with IDT (Ma et al., 2017a). The devices based on PBDB-T: DTNIC8 blend films delivered a PCE of 9.03% with high FF of 73% attributed to well-defined film morphology (Ma et al., 2017b). IOIC2 is a naphthodithiophene-based fused octacyclic acceptor. IOIC2 had larger π-conjugation and stronger electron-rich properties, leading to higher LUMO energy levels, lower bandgap and higher electron mobilities, compared to naphthalene-based fused hexacyclic acceptor. Thus, the devices based on FTAZ: IOIC2 exhibited an excellent PCE of up to 12.3% (Zhu et al., 2018a,b). Another representative fused octacyclic acceptor was CO_i_8DFIC with carbon-oxygen-bridge, which had lower bandgap, higher electron transport properties due to higher electron-donating ability and more planar conjugated structure. When PTB7-Th was used as donor polymer, a high PCE of 12.16% was obtained with high *J*_sc_ of 26.12 mA cm^−2^ (Xiao et al., 2017b). Compared to carbon-bridge, the molecules with nitrogen-bridge usually exhibited stronger electron-donating properties and better solution processing. The INPIC-4F with nitrogen-bridge and fluorinated IC as end group possessed narrow bandgap of 1.39 eV and high electron mobility as well as strong crystallinity. A PCE of 13.13% was achieved with PBDB-T as donor (Sun et al., 2018). To improve electron-donating ability, in addition to increasing the conjugation length, dithienopicenocarbazole (DTPC) possessed strong electron-rich properties by broadening the central core to two-dimensional conjugation system. DTPC-DFIC possessed low band gap of 1.21 eV and exhibited a PCE of 10.21% with PTB7-Th as donor (Yao et al., 2018). A novel small molecular acceptor (BZIC) bearing a D-A-D type thieno [3,2-*b*] pyrrolo-fused pentacyclic benzotrizole core was reported. A broad absorption spectra with optical bandgap of 1.45 eV was achieved due to increased intramolecular electronic interactions from D-A-D conjugated structure. BZIC was the first acceptor material based on a weak electron-deficient unit flanking with electron rich ring as central core rather than electron-donating fused ring unit like IDTT as core. By using HFQx-T as polymer donor, the OSCs exhibited a PCE of 6.30% (Feng et al., 2017b). Most of the above-mentioned acceptor molecules exhibited only one strong absorption band due to strong intramolecular charge transfer. However, M-BNBP4P-1 was developed with two strong absorption bands due to its delocalized LUMO and localized HOMO. The devices showed a PCE of 7.06% with PTB7-Th as donor (Liu et al., 2017a). IID-IC was an A-D-A'-D-A type acceptor. Because of the partially suppressed intramolecular charge transfer effects with the introduction of additional electron-deficient isoindigo unit, IID-IC exhibited a full width at half maximum of 190 nm but only 95 nm for ITIC. The OSCs based on J61: IID-IC delivered a PCE of 2.82% with broad photoresponses from 320 to 780 nm (Miao et al., 2018).

**Table 6:** Summary of the photophysical and photovoltaic properties of fused eptacyclic small molecule acceptors.

**Acceptor**	**Donor**	***E*_g_ (eV)**	**LUMO/HOMO (eV)**	**μ_e_ (cm^2^·V^−1^·s^−1^)**	***V*_oc_ (V)**	***J*_sc_ (mA cm^−2^)**	**FF (%)**	**PCE (%)**
NITI	PBDB-T	1.49	−3.84/−5.68	1.19 × 10^−4^(S,B)	0.86	20.67	71	12.74
NSTI	PBDB-T	1.58	−3.87/−5.54	1.06 × 10^−4^(S,B)	0.83	16.47	75	10.33
IHIC	PTB7-Th	1.38	−3.93/−5.45	1.20 × 10^−3^(S,B)	0.75	19.01	68	9.77
TPTT-IC	PBT1-C	1.63	−3.95/−5.78	3.23 × 10^−4^(S,B)	0.96	15.6	70	10.5
DTNIC8	PBDB-T	1.73	−3.93/−5.91	2.80 × 10^−5^(S,B)	0.96	12.92	73	9.03
IOIC2	FTAZ	1.55	−3.78/−5.41	3.23 × 10^−4^(S,B)	0.90	19.7	69	12.3
CO_i_8DFIC	PTB7-Th	1.26	−3.88/−5.50	3.91 × 10^−5^(S,B)	0.68	26.12	68	12.16
INPIC-4F	PBDB-T	1.39	−3.94/−5.42	5.00 × 10^−4^(S,B)	0.85	21.61	72	13.13
DTPC-DFIC	PTB7-Th	1.21	−4.10/−5.31	3.60 × 10^−4^(S,B)	0.76	21.92	61	10.21
BZIC	HFQx-T	1.45	−3.88/−5.42	8.97 × 10^−5^(S,B)	0.84	12.67	59	6.3
M-BNBP4P-1	PTB7-Th	1.40	−3.93/−5.34	1.47 × 10^−4^(S,B)	0.78	14.62	62	7.06
IID-IC	J61	1.71	−3.95/−5.99	4.15 × 10^−5^(S,B)	0.83	6.36	53	2.82

*S stands for the mobility measured by the space charge limited current (SCLC) method and O for the organic field effect transistor (OFET)method; N stands for the neat film and B for the blended film*.

## Summary and outlook

In this review, two types of promising small-molecule electron acceptors were discussed: PDI based acceptors and A-D-A fused-ring electron acceptors. Traditional PDI units tended to form large aggregate domains leading to low exciton separation and highly torsional PDI derivatives could decrease the charge transport. Thus, a series of strategies, such as: forming PDI dimers and 3D PDI derivatives *etc*., were used to find the balance toward a certain aggregations that exhibited efficient exciton separation without sacrificing charge transfer and mobility. A-D-A type acceptor materials have been developed rapidly and have made exciting progress with highest PCE over 14%. In general, from fused tricyclic to fused octacyclic system, larger central core possessed redshifted absorption and high electron mobilities. Side chains were used to ensure solubility in common solvents and to inhibit strong self-assembly as well as to regulate molecular orientation and morphology. Electron-deficient end groups were used to tuning the LUMO energy level and π-π stacking.

Although non-fullerene-based solar cells have made tremendous progress in recent few years, in order to meet practical applications, designing and synthesizing new acceptor materials, pairing with donor materials, together with technical progress in device fabrications are highly desirable. When designing new active layer materials, basic properties such as absorption, energy levels and charge transport should be carefully considered. Another needed to consider is the cost and stability. We believe that a bright future for realizing high-performance and practical non-fullerene OSCs can be expected.

## Author contributions

ZZ collected the references, drew the structures, wrote the first draft of the manuscript; JY and QW helped with the revision of the manuscript and answered questions the reviewers raised; YZ supervised this project, revised the manuscript and helped all the submissions and giving the answers.

### Conflict of interest statement

The authors declare that the research was conducted in the absence of any commercial or financial relationships that could be construed as a potential conflict of interest.
